# Current Development and Applications of Super-Resolution Ultrasound Imaging

**DOI:** 10.3390/s21072417

**Published:** 2021-04-01

**Authors:** Qiyang Chen, Hyeju Song, Jaesok Yu, Kang Kim

**Affiliations:** 1 Department of Bioengineering, School of Engineering, University of Pittsburgh, Pittsburgh, PA 15261, USA; qic41@pitt.edu; 2 Center for Ultrasound Molecular Imaging and Therapeutics, Department of Medicine, School of Medicine, University of Pittsburgh, Pittsburgh, PA 15261, USA; 3 Department of Robotics Engineering, Daegu Gyeongbuk Institute of Science & Technology (DGIST), Daegu 42988, Korea; hyeju@dgist.ac.kr; 4 DGIST Robotics Research Center, Daegu Gyeongbuk Institute of Science & Technology (DGIST), Daegu 42988, Korea; 5 Division of Cardiology, Department of Medicine, School of Medicine, University of Pittsburgh, Pittsburgh, PA 15261, USA; 6 McGowan Institute of Regenerative Medicine, University of Pittsburgh, Pittsburgh, PA 15219, USA; 7 Department of Mechanical Engineering and Materials Science, School of Engineering, University of Pittsburgh, Pittsburgh, PA 15261, USA

**Keywords:** super-resolution, ultrasound imaging, deep learning, clinical applications

## Abstract

Abnormal changes of the microvasculature are reported to be key evidence of the development of several critical diseases, including cancer, progressive kidney disease, and atherosclerotic plaque. Super-resolution ultrasound imaging is an emerging technology that can identify the microvasculature noninvasively, with unprecedented spatial resolution beyond the acoustic diffraction limit. Therefore, it is a promising approach for diagnosing and monitoring the development of diseases. In this review, we introduce current super-resolution ultrasound imaging approaches and their preclinical applications on different animals and disease models. Future directions and challenges to overcome for clinical translations are also discussed.

## 1. Introduction

Abnormal alterations, including the development, degeneration, and regeneration, of the microvasculature, are reported to be associated with several critical diseases, such as tumor development [1,2,3], progressive kidney disease [4,5,6,7], and the development of atherosclerotic plaque [8,9,10]. Therefore, changes of microvasculature would serve as a useful index for diagnostics and prognostics of such diseases. Several imaging modalities, including microcomputed tomography (micro-CT) [11,12], optical coherence tomography (OCT) [13,14], and magnetic resonance imaging (MRI) [15,16,17,18,19], have been employed successfully in preclinical studies to image the changes of microvasculature inside target organs. Although these imaging methods achieved a high spatial resolution, they have their own their own limitations. Micro-CT is limited by hazardous radiation and contrast agents, while OCT suffers relatively poor imaging depth. As for MRI, imaging systems are bulky and costly, which hinder widespread or repeated applications. Ultrasound imaging that has the advantage of safety, noninvasiveness, portability, affordability, and ease of use, has been explored as a potential approach for imaging microvessels. Conventional noninvasive ultrasound imaging methods for imaging vessels mainly include Doppler ultrasound imaging [20] and contrast-enhanced ultrasound (CEU) imaging [21,22,23,24,25]. However, neither of these techniques provides sufficient spatial resolution for assessing microvessels, mainly due to the acoustic diffraction limit, which is half of the wavelength of the operation ultrasound frequency. Therefore, an ultrasound imaging technique that can achieve spatial resolution beyond the acoustic diffraction limit would be encouraging for broad use in clinics for diseases that are associated with abnormal alterations in the microvasculature.

In 2006, optical super-resolution imaging techniques, including fluorescence photoactivated localization microscopy [26], photoactivated localization microscopy (PALM) [27], and stochastic optical reconstruction microscopy (STORM) [28], were first introduced. The basic idea of super-resolution imaging is to localize the centroid of each randomly blinking fluorescence source based on the system point spread function (PSF). The location information of each blinking fluorophore was stacked up over a substantial sequential dataset that was captured by a fast camera to form an image spatially resolved in subwavelength resolution. By this approach, a spatial resolution down to tens of nanometers was achieved. Inspired by optical super-resolution imaging techniques, super-resolution ultrasound (SRU) imaging was introduced [29,30,31,32,33] for noninvasive imaging of microvasculature by using ultrasound contrast agents that travel through vascular network to replace the role of fluorophores in optical super-resolution imaging. While spatial resolution was sacrificed compared to optical super-resolution, due to the limit of ultrasound operating frequency, the ultrasound approach achieved a larger imaging depth. The technology components of SRU imaging mainly consist of ultrafast, ultrasound imaging [34], a state-of-the-art clutter filter [35] that extracts microbubble signals, and novel microbubble localization algorithms [29,30,31,32,33,36,37] that pinpoint the original locations of the microbubbles. The developed SRU imaging technologies have been successfully tested in preclinical studies with different animal and disease models, which demonstrated great potential for future clinical applications. Some representative conventional technical approaches of SRU, and their in vivo applications, were well described and summarized in a recent review paper [38]. In addition to these conventional SRU approaches, a deep learning approach has recently been adopted for SRU imaging [39,40,41,42]. In this review, we introduce the different SRU imaging approaches, especially the deep learning approach, and summarize current preclinical studies in different disease models that have been successfully performed. The vision for future clinical applications, and the major challenges for SRU imaging to overcome, are also discussed.

## 2. General Technical Components of Super-Resolution Ultrasound Imaging

**Figure 1:**
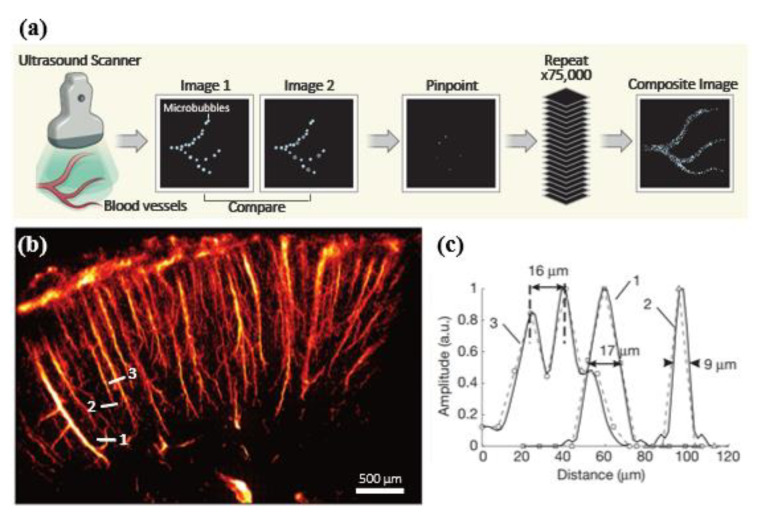
(**a**) An illustration of the concept of SRU imaging (reprinted with permission from Ref. [44]. Copyright 2015 Springer Nature). (**b**) The reconstructed super-resolved brain microvasculature with the resolution of λ/10 [29]. (**c**) Interpolated profiles along the marked lines (reprinted with permission from Ref. [29]. Copyright 2015 Springer Nature).

Figure 1a illustrates the overall block diagram of SRU imaging. The blinking fluorescence sources in the photoactivated localization microscopy can be replaced with microbubbles, used for the contrast agent of ultrasound in SRU imaging, as the spatial locations of microbubbles in the bloodstream are stochastically changed. The following localization technique is a method to find the centroid of the single microbubble signal, localizing each point source with subwavelength precision. Throughout this process, spatial resolution can be improved by up to one-tenth of the wavelength, theoretically [29]. Note that the acoustic response to the point source can be estimated to the PSF of the imaging system. Therefore, the extraction of a single microbubble signal from the original image data is an essential key component for implementing SRU imaging. The first trial for decluttering is a subtraction between neighboring frames to remove the stationary tissue component and maintain moving microbubble signals [32]. Other clutter filtering techniques used in Doppler imaging are also studied for decluttering purpose. Researchers show that the combination of the singular value decomposition-based adaptive clutter filter and a large number of the spatiotemporal image sets, with the ultrafast imaging, would outperform traditional infinite impulse response (IIR) filter-based clutter filtering techniques [35]. This method decomposes the large-sized elongated skinny matrix into the spatial and temporal basis vector matrixes and a diagonal eigenvalue matrix-weighting factor. The combination of these decomposed vectors represents several components of the images, such as stationary tissue, slow-moving tissue, fast-moving particle-microbubbles, and randomly varied value noise. Therefore, microbubbles signals could be exclusively extracted from the images with an adequately selected rank of vector-matrix combinations. The signals, other than the selected ranks, are then removed to maintain only the valid microbubble signals.

**Figure 2:**
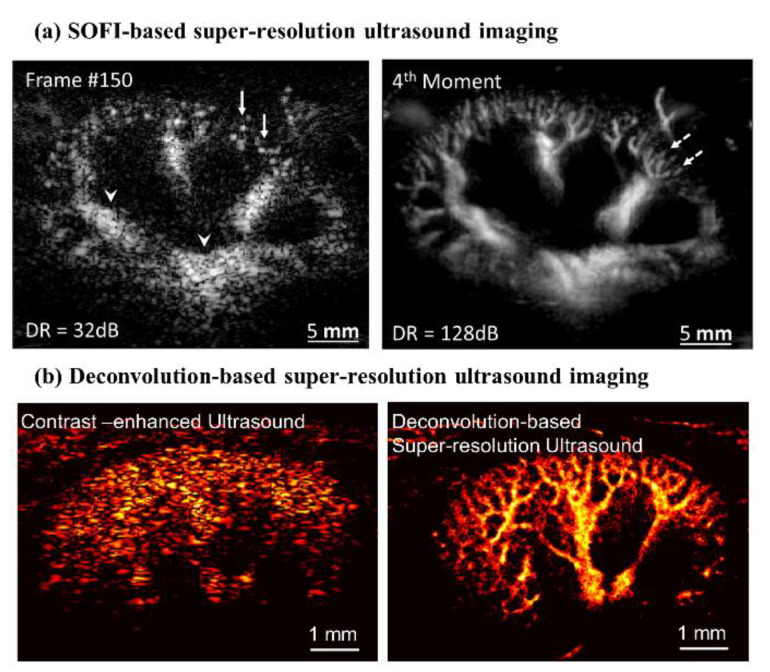
(**a**) SOFI-based super-resolution ultrasound imaging—left panel shows a frame of B-mode; right panel shows the reconstructed image. (Reprinted with permission from Ref. [43]. Copyright 2017 IEEE). (**b**) Deconvolution-based super-resolution ultrasound imaging—left panel shows a frame of B-mode; right panel shows the reconstructed image. (Reprinted with permission from Ref. [45]. Copyright 2020 Elsevier).

The next core component is a method seeking the point source location. Each microbubble location can be precisely localized in subwavelength resolution by fitting with the predetermined PSF of the imaging system [29]. This method achieves the spatial resolution up to 10 microns (≈λ/10, with a custom-made 128-element linear array transducer centered at 15 MHz), which is further beyond the acoustic diffraction limit, as shown in Figure 1c [29]. Note that the imaging system’s PSF is assumed as the fixed two-dimensional Gaussian function determined by the transmit wave characteristic. However, this approach requires a huge number of the dataset, 75,000 frames (150 s), for a single super-resolved image in this study. Long data acquisition is the main drawback for clinical applications, except imaging the brain, which can be possibly fixed in position during the scan period due to the motion artifact. In the following studies, therefore, several groups suggest techniques in efforts to improve temporal resolution. These include Super-Resolution Optical Fluctuation Imaging (SOFI)-based [43] and deconvolution-based [36] SRU imaging technologies to broaden the clinical applications to other organs and diseases, as shown in Figure 2 [36].

SOFI-based super-resolution imaging uses a relatively high concentration of microbubbles, while the traditional super-resolution method utilizes a diluted concentration for better separation of microbubbles [43]. Bar-Zion et al. [43] suggest a parametric model of the contrast-enhanced ultrasound signal of microbubbles to quantify the volume cell, instead of counting the number of microbubbles. High order statistics calculations could improve spatial resolution by 60% at the 4th moment, as shown in Figure 2a, using a L15-4 linear array transducer. They successfully demonstrate their super-resolution imaging approach using a rabbit kidney tumor model with only 150 frames of data, which allows for a 500-times faster scan time than the prior method. The SOFI-based method that utilized high order statistical computations [43] achieved higher temporal resolution than the previous ultrasound localization microscopy [29]. However, spatial resolution and the signal-to-noise ratio were compromised because the dynamic range of image intensity increased as the higher-order statistics were used. Some researchers suggest using a nonlocal means (NLM) denoising filter on the spatiotemporal domain to remove noise from the background signal, while preserving the signal from flowing microbubbles [37]. It should be noted that use of the spatial domain filter to eliminate noise is, in general, challenging, as shown in the previous study, as the amplitude of the background noise looks very similar to signals from microbubbles. They then applied bipartite graph-based microbubble tracking, with persistence control for enhanced microbubble signal quality and tracking fidelity. The localized microbubbles located in each frame could be paired with, and followed by, microbubbles at adjacent frames. When using a 128-element linear array transducer, centered at 8 MHz, vessels as small as 57 µm at depth of 2 cm were reconstructed. Moreover, microvessels 76 µm apart were distinguished in a rabbit kidney in vivo.

Another trial to improve temporal resolution is the employment of deconvolution and spatiotemporal-interframe-correlation (STIC) data acquisition techniques [36]. Regardless of the local density of the microbubbles, the deconvolution approach can localize each microbubble location from the clumped microbubble signal. It therefore enables the utilization of all acquired frames. Note that clumped microbubble signals have to be discarded in other approaches, resulting in a long scan time. Therefore, deconvolution-based SRU imaging only uses 300 image frames to reconstruct a single super-resolved ultrasound image while maintaining a spatial resolution of 41 μm; that is, 1/5 of the wavelength with a 128-element linear array transducer centered at 7.7 MHz. The calculation complexity of the deconvolution method [36] is lower than the above methods while it utilizes the iteration procedure. However, many physiological events are still faster than the data acquisition speed with a deconvolution of 0.6 s. Researchers implemented the STIC data acquisition technique, that was used for the 3D fetal cardiography, to capture rapid physiological events [46]. STIC algorithms allow for a realignment of sequentially acquired image data based on the reference signal, such as the cardiac pulsation, to make use of more frames that are synchronized. Figure 2b shows typical SRU images in an in vivo acute kidney injury in a mouse model, demonstrating the clinical feasibility for kidney applications [37,45]. Furthermore, the implemented SRU imaging technologies were further validated by comparison with micro-CT images in the following study [45]. 

One main challenge of super-resolution ultrasound imaging technology is maintaining the spatial and temporal resolution at the same time. Ultrasound localization microscopy achieved the spatial resolution of 1/10 of the wavelength beyond the acoustic diffraction limit. However, this method scarifies the temporal resolution, as the huge dataset, ~75,000 frames, corresponding to data acquisition time of 150 s, are required to track every flowing individual microbubble [29]. The SOFI-based method achieved a higher temporal resolution of ~150 frames, and a data acquisition time of 0.3 s-, with the time-dependent statistics of the microbubbles [43]. It also increased the spatial resolution around a factor of $\sqrt{2}$, which provides further fine spatial resolution beyond the acoustic diffraction limit. The deconvolution-based super-resolution ultrasound imaging method offers compromised spatial and temporal resolutions between the ultrasound localized microscopy and the SOFI approach. The spatial resolution of 1/5 of the wavelength and the temporal resolution of 0.6 s (~300 frames) were achieved [36]. Depending on their advantages, these methods could be applied for different applications. The overall performances of the representative SRU imaging technologies are compared in Table 1.

## 3. Deep Learning-Based Super-Resolution Ultrasound Imaging

A single super-resolved image can be only reconstructed with a sufficient number of frames of the localized microbubbles for a sufficient signal-to-noise ratio. This large-scale data acquisition results in a relatively long scan time, which may introduce potential motion artifacts. The low consistency of tissue caused by motion could decrease localization accuracy. Thus, a practical limitation of SRU imaging for clinical translation is the trade-off between data acquisition time and localization accuracy. The deep learning-based approach has demonstrated promising achievements, both in temporal accuracy and in reconstruction accuracy, when using a relatively high-concentration microbubble injection.

**Figure 3:**
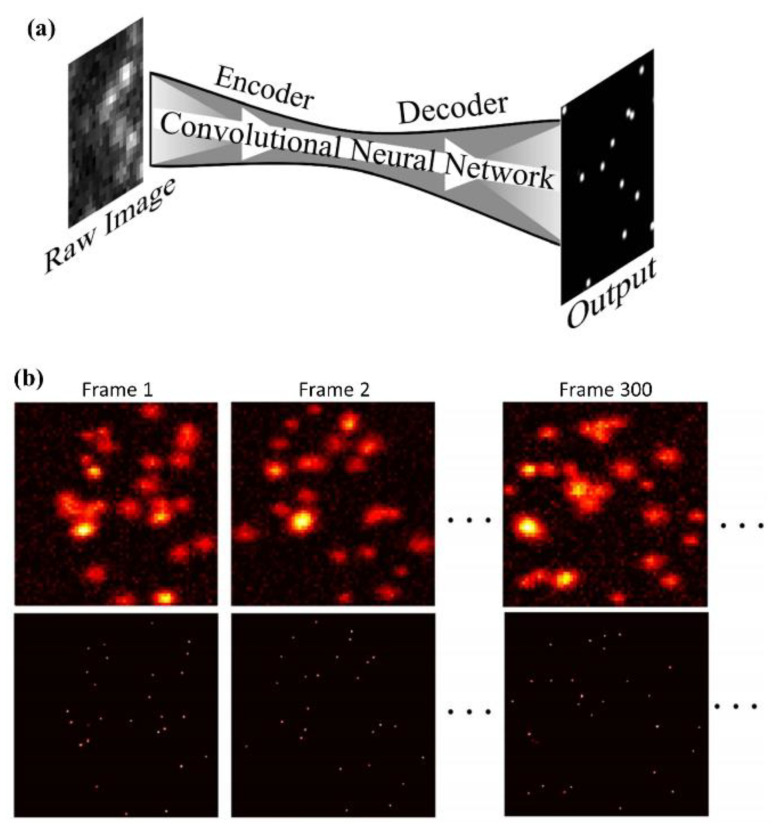
Super-resolution ultrasound localization microscopy through deep learning. (**a**) U-net based architecture for Deep-ULM [39], mSPCN-ULM [40], and a multiple-targets detecting network [47]. (Reprinted with permission from Ref. [47]. Copyright 2018 OSA) (**b**) An example of deep learning-based localization techniques—translation from the low-resolution image (top panel) to high-resolution frames (bottom panel) by the deep learning network [40,49]. (Reprinted with permission from Ref. [40]. Copyright 2020 IEEE).

The deep learning-based ultrasound localization microscopy (Deep-ULM) is the first trial to let artificial intelligence separate individual microbubble signals from the dense microbubble cloud signal [39]. An increased concentration of microbubbles would reduce overall data acquisition time. The Deep-ULM, inspired by the deep learning network for super-resolution stochastic optical-resolution microscopy (Deep-STORM), adopts a network based on the fully convolutional U-net, performing the nonlinear end-to-end mapping between low-resolution input frames to high-resolution outputs, as shown in Figure 3 [39,47,48,49]. For the synthetic training dataset, randomly located microbubble positions were generated first. The diameters of the microbubbles were also randomly determined, making them similar to the actual microbubble signals. Then, the convolution between the simulated microbubbles and the point spread function can work as a synthetic low-resolution ultrasound image. Then, the simulated ultrasound images were paired with the actual locations of the microbubbles for training through the network. The encoder extracted the dense and aggregated features from low-resolution images. In the decoding layers, the features extracted in the encoding layers were upsampled and deconvolved to align the value for high-resolution image reconstruction. After the training, the trained mapping process reconstructs the low-resolution ultrasound B-mode image to a high-resolution image through feature extraction and upsampling. Besides, the Deep-ULM reduces the computational complexity with the GPU acceleration, allowing it to resolve 1250 high-resolution patches of 128 × 128 pixels within a second. Therefore, the model-based approach has great benefit and potential for implementing the real-time imaging system. 

Several modified or alternative network structures have been studied for a faster data processing time in the following research. For example, the convolutional neural network (CNN) was suggested for identifying individual scatters using high concentration microbubbles [50]. This network has a similar structure to U-net and is composed of an encoder–decoder with pooling and un-pooling, but without skip connections. Unlike the previous deep learning-based ULM method, the ground truth data were acquired after the radio frequency (RF) dataset was simulated. The binary confidence maps obtained through the simulated RF dataset can generate the ground truth data. In addition, the RF signals were not beamformed with the delay-and-sum algorithm, but delayed and sampled to have the same number of confidence maps along the axial direction. Considering that the training dataset is a key factor for trained network performance, it is worth more than passing attention to the improvement of deep learning networks based on a precisely tailored synthetic dataset. 

**Figure 4:**
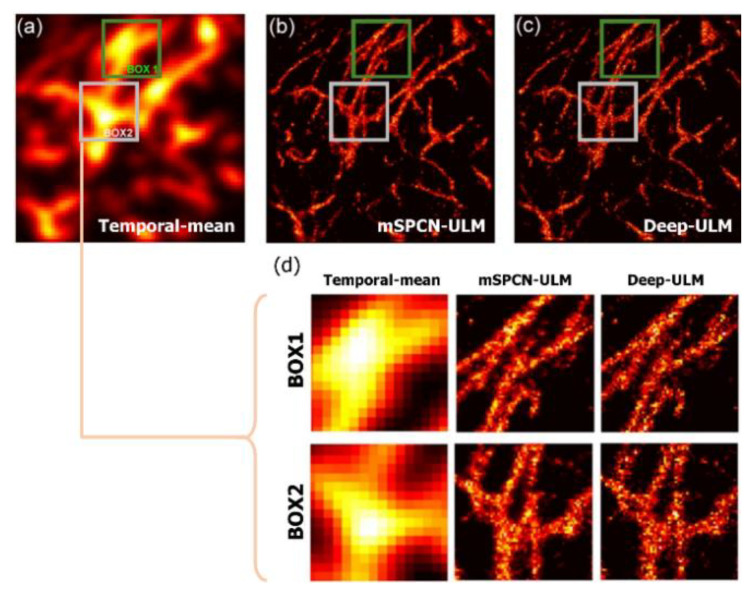
Comparison between mSPCN-ULM [40] and Deep-ULM [39]. (**a**) The temporal–mean image was obtained by averaging all the US images. (**b**) Super-resolution images obtained by the mSPCN-ULM. (**c**) Deep-ULM. (**d**) The magnified sections of (**a**–**c**) [40]. (Reprinted with permission from Ref. [40]. Copyright 2020 IEEE).

Recently, modified subpixel convolutional neural network (mSPCN) architecture with residual blocks has been suggested for optimization without exhausted parameter tuning and fast data processing speed [40,41,42]. The subpixel convolutional neural network architecture in the mSPCN-ULM decodes features with a trained upscaling filter and reduces computational complexity. Moreover, residual learning allows an increase in the network depth without losing gradient, and improves training accuracy without a parameter tuning process, as shown in Figure 4. The mSPCN-ULM showed increased temporal resolution using a higher microbubble concentration compared with the above deep learning techniques. The training process was conducted using the synthetic data created in the same way as the previous Deep-ULM method presented. However, further performance degradation is expected in vivo cases since the discrepancy between the synthetic training dataset and the real in vivo data would become more extensive due to several artifacts and nonlinear responses.

Deep learning technology is also applied in another applications, such as the decluttering process [51]. The extraction of small microbubble signals from only a few pixels in the image with noise is challenging. Researchers have employed a 3D convolutional neural network (3D-CNN) to solve this problem. This network has been known as an optimal network for human action recognition in airport surveillance video sequences, which is similar to flowing microbubble detection in the B-mode image sequence (2D + 1D). The proposed method’s performance is comparable to the use of singular value decomposition (SVD) filtering in conventional SRU imaging sequences with a lightweight computational burden. 

So far, deep learning networks trained with the noised synthetic data showed a resolution comparable to existing SRU imaging. However, the number of in vivo studies with deep learning are limited so far. Therefore, further investigations, especially in vivo evaluations, of deep learning applications should follow. Below, Table 2 shows an overall comparison of the representative deep learning-based technologies currently used in SRU imaging.

## 4. Current Biomedical Applications of Super-Resolution Ultrasound Imaging

With unprecedented spatial resolution and practically reasonable temporal resolution achieved, SRU could be a promising diagnostic tool for diseases associated with abnormal vascular alterations. It also has the potential to be a preferred approach for monitoring disease progression and therapeutic efficacy due to its noninvasiveness, low cost, safety, and widespread accessibility. In this section, some representative applications of SRU in preclinical studies on different organs and disease models, and a very limited first-in-human use, are introduced and discussed.

### 4.1. Cancer

Cancer is the second leading cause of death in the world [52]. Early detection of malignant lesions can greatly increase the chances of successful treatment [53]. One of the early changes that can differentiate cancer from normal tissues is malignant angiogenesis, which has been recognized as an important biomarker for cancer diagnostics [2,54]. The features of the microvascular network associated with malignant tumors, including density, branching, size, and inhomogeneity, have been observed to be abnormal compared to that of healthy tissue [3,55,56,57,58]. In past decades, superharmonic contrast ultrasound imaging, also known as acoustic angiography, has been utilized to visualizing the microvasculature and detect the morphology abnormalities associated with tumor-induced angiogenesis in vivo [59,60,61,62,63,64]. However, the performance of this imaging technique suffered mainly from the limit of the spatial resolution constrained by the acoustic diffraction limit of the operating ultrasound frequency. An SRU that can overcome this limitation has been explored to detect microvascular changes at much higher resolution and sensitivity, both at an early stage and during tumor progression.

For demonstrating the proof-of-concept and further improving SRU technology, animal tumor models have been adopted in several in vivo studies [43,65,66]. In 2016, Lin et al. successfully imaged the subcutaneous fibrosarcoma tumors implanted in a rat in vivo, with a ten-fold resolution improvement compared to conventional ultrasound imaging by using SRU. This study demonstrated the imaging capability of SRU and the potential of characterizing a tumor-associated microvascular angiogenesis [65]. In the following study, their group evaluated the sensitivity of SRU imaging on the same rat tumor model using microbubbles of different sizes, and showed the sensitivity improvement by using larger microbubbles. For the purpose of shortening the scan time, Bar-Zion et al. proposed an SRU imaging technique with a methodology that was used in super-resolution optical fluctuation imaging (SOFI). This technology was tested by imaging the vasculature around and inside the hind-limb intramuscular VX-2 tumor, and the improvement in temporal resolution was presented [43]. 

**Figure 5:**
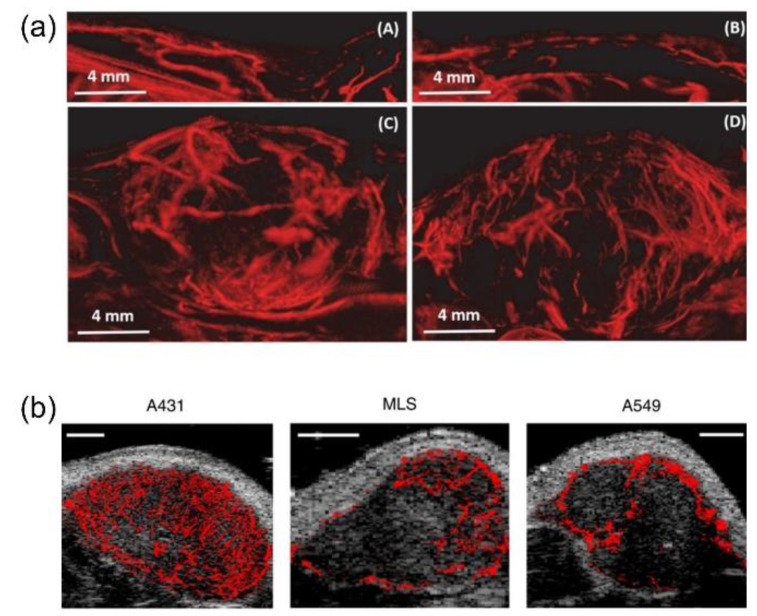
Representative SRU images of microvasculature in tumor-bearing tissues. (**a**) Maximum intensity projections of 3D SRU imaging on healthy rats (upper panel), and tumor-bearing rats (lower panel). (Reprinted with permission from Ref. [67]. Copyright 2017 Ivyspring International Publisher) (**b**) SRU imaging of tumors with different vascular phenotypes. (Reprinted with permission from Ref. [68]. Copyright 2018 Springer Nature).

After demonstrating the proof-of-concept in tumor microvasculature imaging, preclinical studies have been conducted to examine the capability of SRU for tumor diagnosis. Lin et al. performed SRU imaging in three dimensions on tumor-bearing rats implanted with subcutaneous fibrosarcoma and compared the microvascular features with the healthy rats [67]. An L11-5 linear probe (Verasonics Inc., Redmond, WA, USA) was mounted to a motorized precision motion stage synchronized with the imaging system to perform the 3D scan. The reconstructed microvascular images by SRU showed a greatly improved spatial resolution compared to the traditional acoustic angiography. As shown in Figure 5a from the study, vessels in the tumor-bearing tissues had a higher tortuosity compared to the control, which implied tumor-associated microvascular angiogenesis. The results demonstrated the potential of differentiating diseased and healthy tissues by evaluating vascular structure using SRU imaging. With the help of the fine details of the vasculature network provided by SRU technology, the capability of SRU for discriminating different tumor types was also shown by the study of Opacic et al. [68]. The fine vascular networks in tumors with different vascular phenotypes were reconstructed by motion model SRU imaging (Figure 5b). Functional parameters, including relative blood volume (rBV), blood flow direction, blood flow velocity, distances to vessels, distances, and velocities, were able to be derived by SRU imaging, and utilized to differentiate different tumor types with the verification of histology successfully. 

**Figure 6:**
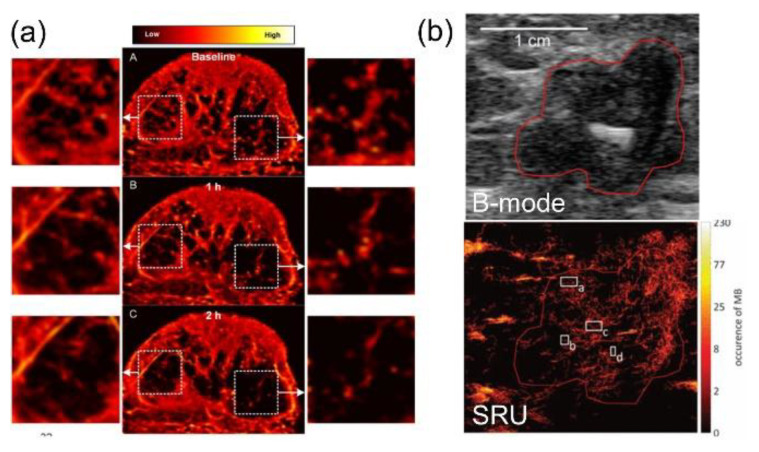
Representative SRU images of microvasculature from (**a**) breast cancer-bearing mice in response to the treatment (Reprinted with permission from Ref. [70]. Copyright 2017 IEEE), and (**b**) the patient with triple-negative breast carcinoma. (Reprinted with permission from Ref. [71]. Copyright 2019 IEEE).

SRU imaging was further evaluated on specific cancer types, which is the pathway towards clinical translation. Breast cancer, which is the most common type of cancer in women, with the second highest mortality rate [69], is also one focused area for the applications of SRU. Ghost et al. applied SRU imaging to longitudinally monitor changes in the tumor microvascular network of triple-negative breast cancer-bearing mice in response to the treatment [70]. The vessel-to-tissue ratio of the tumor tissue was found decreased progressively after the tumor-targeted therapeutic (Figure 6a), which was consistent with the immunohistological findings. This study suggested the potential of in vivo SRU imaging for monitoring early tumor response to drug treatment. Clinical pilot studies of SRU imaging on patients with breast cancer was further conducted by the Schmitz group [68,71]. Motion model SRU imaging was performed on patients with breast cancer after treatment with first, second, and third cycles of neoadjuvant chemotherapy. By SRU imaging, improved spatial resolution and functional information, including flow velocities, could be derived (Figure 6b) [71], which outperform conventional CEU imaging. The increase in rBV of the tumor tissue and the decrease in tumor size were found after the treatment [68]. The studies could be a scheme for further extended clinical studies and to promote future clinical applications.

In general, as one of the key features of a tumor is a dense microvasculature network, the tumor model would serve as a good candidate for demonstrating the imaging capability of SRU and for validating the technical improvements with new approaches for SRU. For the potential applications of SRU imaging on cancer, the studies mentioned above show that, with the fine structure of the microvascular networks reconstructed by SRU, several functional parameters can be accurately derived to help diagnose malignant tumors or differentiate different tumor types. Several studies have been performed, specifically on breast cancer, and reported promising results. Experiments on human subjects were also initiated. Extended clinical studies in the near future are expected for the clinical translation of this technology.

### 4.2. Kidney

Chronic kidney disease (CKD), which has a high incidence rate among adults [72,73], is typically induced by several risk factors, including diabetes, high blood pressure, heart disease, an episode of acute kidney injury, etc. [72,74]. One mechanism for the progression of CKD is the degradation of the renal microvasculature and perfusion impairment [75,76]. Therefore, the detection of renal microvascular changes would be of great importance for the early diagnosis and monitoring of CKD. However, diagnostic tools that enable noninvasive diagnostics and monitoring of renal microvascular alterations during progressive kidney disease are still lacking. Conventional ultrasound imaging techniques, including contrast-enhanced ultrasound imaging [21,22] and Doppler ultrasound imaging [20], have been explored to evaluate microvascular changes during the disease’s progression. However, the spatial resolution is not ideal, mainly due to its insufficient sensitivity and the acoustic diffraction limit [77]. The emerging SRU imaging technique would be a promising approach to overcoming these barriers.

**Figure 7:**
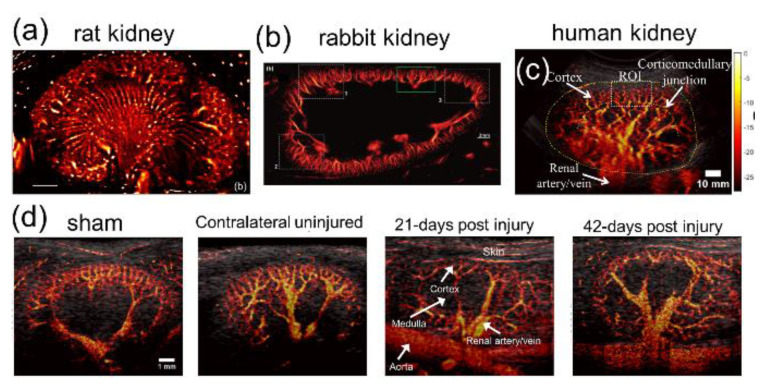
Representative SRU images of microvasculature from rat kidney (Reprinted with permission from [78], Copyright 2017 Springer Nature) (**a**); rabbit kidney using Kalman filter-based method (Reprinted with permission from Ref. [79]. Copyright 2020 IEEE) (**b**); healthy human kidney (**c**); mouse kidney in group of sham, contralateral uninjured, 21-days after ischemia–reperfusion injury, and 42-days post injury (Reprinted with permission from Ref. [45]. Copyright 2020 Elsevier) (**d**).

Several studies have already been successfully conducted on animal kidneys in vivo to show the capability, as well as the technical improvements, of imaging the renal microvasculature by SRU [37,43,78,79]. Foiret et al. depicted the microvascular structure and characterized the vessels with a flow rate below 2 mm/s of rat kidney, with Contrast Pulse Sequencing (CPS) mode using a 6.9 MHz ultrasound probe (CL15-7, Phillips ATL, MA, USA) [78]. Song et al. proposed the spatiotemporal NLM denoising method, together with the bipartite graph microbubble pairing and tracking method, and showed improved performance of SRU on rabbit kidney [37]. With an operating frequency of 8 MHz and mechanical index of 0.4, a single renal microvessel as small as 57 μm was identified, and microvessels that were 76 μm apart were clearly separated. Their group further developed the Kalman filter-based SRU method and presented a robust measurement of the renal microvascular flow with reduced MB events in the rabbit kidney [79]. Figure 7a,b shows the representative SRU images of the renal vascular network in rat and rabbit kidneys from the studies mentioned above. 

On the way towards the clinical application of SRU imaging on kidneys, more affirmative data from studies on clinically relevant animal models are required. Yang et al. performed SRU imaging on an acute ischemic–reperfused rat kidney and a normal rat kidney to investigate the in vivo feasibility of evaluating microvascular changes during progressive kidney disease. The results showed that the blood flow speed in the injured rat kidney (<10 mm/s) was much lower than that in the healthy kidney (~30 mm/s) [80]. Similar results was achieved by Andersen et al., suggesting that blood flow in the renal microvasculature was measured to be slower after ischemia and reperfusion by SRU imaging [81]. Studies that demonstrated the feasibility of SRU for identifying microvascular alterations during the disease progression, with a larger group of animals and histological verifications, were performed by Chen et al. [45]. In the study, SRU imaging was performed in vivo on mouse kidneys of four different groups (n = 5), including control, kidneys post 21 days of ischemia–reperfusion injury, and kidneys post 42 days of injury (Figure 7d), followed by the histological analysis with a CD31 stain. The results showed that SRU imaging was able to identify renal microvessels as small as 32 μm (<1/3 λ at the frequency of 15 MHz) in vivo and allow for quantification of the changes in kidney morphology and vasculature, including size, rBV, vessel density, and tortuosity, during the progression of the kidney injury. Changes in renal vascular density in the corticomedullary area were validated by a CD31 stain, and a relatively strong correlation was found between SRU and histological measurement. While the former two studies focused more on the change in flow velocity, the latter only examined the features derived from structural information. Future studies that investigate both structural and flow information on the groups of animals in disease models is highly sought.

The next step towards the future translation of SRU imaging on kidneys would be studies on human subjects, including healthy and CKD patients. Studies have been initiated and some preliminary results have already been presented at conferences (Figure 7c) [82]. Motion artifact is one of the major issues to address before successful future translations of kidney SRU imaging, since the breathing motion affects the locations of the organs in the abdomen significantly. In the animal studies, most of the groups applied block matching algorithms on the envelope of B-mode data to estimate and correct the translational breathing motion in lateral and axial directions [37,45,79,80,81]. Foiret et al. utilized linear optimization to better correct both translational and rotational motion [78]. However, out-of-plane motion remains a problem for 2D kidney imaging by using the 1D array transducer. Moreover, breathing motion during human kidney imaging can be more critical compared to experiments on anesthetized animals. A short scan time might offer a practical solution so that an SRU imaging session can be completed with minimized motion artifacts while a human subject holds their breath. In addition, further improvements that enhance MB signals in depth, in clinical abdominal imaging conditions and extended experiments on a larger group of human subjects, are required for future clinical translations of SRU imaging. 

### 4.3. Other Applications

SRU was also applied to other organs or animal models, such as the brain, the femoral artery with atherosclerotic plaque (AP), etc. 

**Figure 8:**
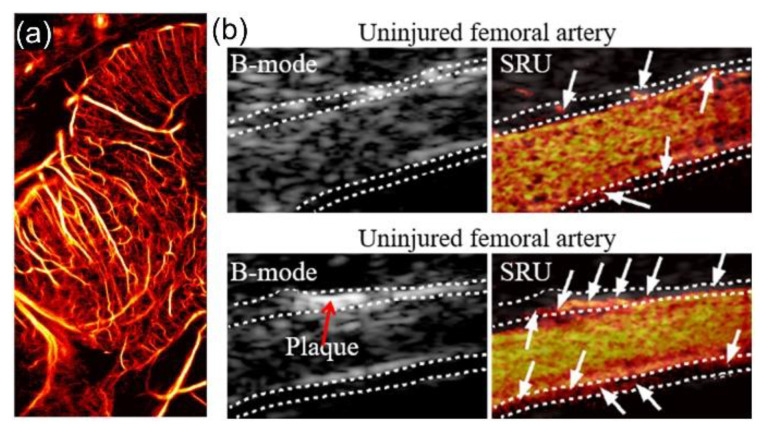
Representative SRU images of microvasculature in chicken embryo brain (Reprinted with permission from Ref. [86]. Copyright 2020 Springer Nature) (**a**), and VV in uninjured (upper panel) and injured (lower panel) rabbit femoral arteries (Reprinted with permission from Ref. [90]. Copyright 2020 IEEE) (**b**).

The pathological process of the small vessels in the brain has been recognized as a contributor to cognitive impairment and dementia [83,84,85]. Therefore, an imaging tool that can resolve small vessels in the brain would be beneficial for the diagnostics and therapeutics of such neurological diseases. Errico et al. initiated a study of brain microvasculature imaging using an SRU technique in 2015 (Figure 1c) [29]. Rat brain microvasculature was imaged with a 15 MHz ultrasound probe through the thinned skull. Vessels as small as 9 μm (1/10 λ) were resolved and the in-plane blood flow profile was achieved, although the scan time was quite long (150 s). Recently, Huang et al. proposed a method that separates spatially overlapping MB events into subpopulations based on spatiotemporal differences in flow dynamics, and successfully visualized chicken embryo brain vasculature with a shortened scan time (~17s) (Figure 8a) [86]. Compared to the kidney imaging, a relatively long scan time could be acceptable for brain imaging, for which the physiologic motions are less pronounced if the ultrasound transducer is fixed to the head. For the future clinical translation of SRU in brain imaging, attenuation and aberration from the skull that significantly degrades imaging performance remains a big challenge which requires further investigation. 

Another potential application of SRU is to monitor the development of atherosclerotic plaque (AP) and predict AP rupture by imaging vasa vasorum (VV) near major vessels. It has been reported that abnormal proliferation of VV and the infiltration into the AP core is key evidence of AP progression and vulnerability [10,87,88,89]. Due to the tiny size of VV, it is challenging to imaging VV in vivo. In a pilot study, Yu et al. successfully identified VV in a rabbit AP in vivo, with the spatial resolution of 45 μm, by the deconvolution-based SRU imaging technique (Figure 8b) [36]. In the follow-up study by the group, the abnormal proliferation of VV near the rabbit femoral artery that was identified by SRU was further validated with subsequent histology and ex vivo microcomputed tomography (µCT), histopathology, and morphology [90]. The experiment protocols and results would encourage extended preclinical studies with larger group of animals and potential human studies in the future. 

Besides the in vivo studies mentioned above, promising results of microvascular imaging by SRU were also achieved in vivo in organs, including rabbit lymph nodes [91], rabbit eyeballs [92], mouse liver [93], and human tibialis anterior muscles [94], which may contribute to broader applications of SRU imaging.

## 5. Limitations and Future Directions 

As we discussed above, SRU imaging has been continuously improved and applied in vivo for different applications in animal models and very limited human uses. However, there are still several major limitations that hinder the eventual clinical translations of this novel technology. 

One of the major limitations is the relatively long scan time. To reconstruct a single SRU image, the accumulation of a large-scale microbubble backscatter of signals is required, which results in a long acquisition time. A typical SRU algorithm that localizes the center of the spatially isolated MBs requires a scan time of several minutes to collect the necessary data [29]. Since SRU imaging is highly sensitive to motion artifacts, the image quality will be notably degraded due to physiological or externally induced motion, which is inevitable during a long scan time. Considering the freehand scan that would be practiced in future clinical applications, the long scan time would be a big barrier. Harput et al. [94] suggested a two-stage motion correction algorithm which calculates the combination of affine and nonrigid image registrations through the motion estimation from the B-mode image, and corrects the motion artifacts of CEU image. The rigid motion artifact correction using a phase-correlation technique was also suggested to remove the blurring caused by subwavelength motions [95]. Although motion correction can help mitigate the motion artifacts [94,95], it still cannot perfectly solve the problem of motion in real cases, which combines rigid motion, nonrigid motion, and out-of-plane motions. Some algorithms that can shorten the scan time have been developed [36,86,92,96], but the spatial resolution is more or less compromised compared to the algorithm that localizes the isolated MBs. The large-scale data will also require a high computation cost for image reconstruction. Utilization of a GPU for data processing could be a solution to reduce the computation load and to possibly realize SRU image processing in real-time in the future. In some studies of SRU imaging using a matrix array, a GPU has already been successfully applied to significantly shorten the computation time for large-scale data set [97,98,99]. 

Another limitation is that image quality is highly dependent on MB concentration and distribution in the blood vessels [77,100]. A low concentration in the vessels will lead to a long scan time and low signal contrast, while a high concentration will degrade localization accuracy and, thus, the spatial resolution of the image. Since MBs are systematically administrated to the blood vessels, it would be difficult to control the real MB concentration in the target area in the clinical practice. Moreover, the dosage may need to be adjusted for different applications in order to have appropriate MB concentration in vessels of the target organs. Deep learning approaches would be a promising direction to overcome this challenge while further evaluations are needed. Moreover, in order to make the technology for broader application in clinics, with no possible safety concerns for some populations, including pregnant women or subjects potentially allergic to microbubbles etc., it is ideal to develop a contrast-agent-free super-resolution. However, no fully developed approaches with ideal performance have been reported so far, except some pioneering initiative works presented in recent conferences [101].

Currently, most in vivo clinical studies of super-resolution ultrasound imaging techniques are conducted on a 2D B-scan with a 1-D array ultrasound transducer. However, 2D cross sectional imaging has limited resolution in the elevational direction, determined by the relatively large elevational beamwidth. The 3D SRU imaging techniques using 2D array arranged 1024 elements in 32 × 32 matrix showed a promising in vitro study result, with the subwavelength resolution in both lateral and elevational directions [97]. The use of a fully sampled 2D matrix array, however, is suffering from the heavy computational complexities of handling huge volumetric data. Several approaches, including (1) an FPGA–GPU structure-based ultrasound system [102], (2) frequency domain beamforming [103], and (3) novel transducer configurations such as the sparse array [98] and the row-column array [99], are suggested to overcome such limitations. An FPGA–GPU structure-based ultrasound system could manage the huge data size because it benefits from both the FPGA, for high-speed data transfer, and the GPU for processing [102]. Another study performs the beamforming of 3D volumetric imaging in the frequency domain to reduce computational complexity [103]. In addition to these approaches, the sparse array [98]-based volumetric super-resolution ultrasound imaging technique utilizes half of the channels compared to the fully sampled array and achieves a comparable resolution [104]. Further research on sparse array-based super-resolution ultrasound imaging should continue to deal with the grating lobe [99] and the fastidious optimization process [104] of the sparse array [105]. The other approach, the row–column array-based super-resolution imaging method [99], reduces the number of connections from N^2^ to 2N in the N × N 2D array by utilizing two orthogonal arrays [104]. Although the resolution of the row–column array method is comparable, edge artifacts caused by the long element should be suppressed with mechanical apodization [106].

Besides, for abdominal, transcranial, and brain applications in humans, the tradeoff between spatial resolution and penetration depth needs to be considered; the imaging depth will be significantly larger in human subjects. It will be more challenging to detect microbubbles in the microvessels at deep depth due to acoustic attenuation. Techniques that can enhance the transmitted energy without destroying MBs, such as coded excitation [107,108,109,110], may be one potential solution. Utilization of microbubbles with larger sizes may also help enhance signals, as reported in the previous study [66].

## 6. Conclusions

In the past decade, SRU imaging technology that can achieve microvascular images with spatial resolution beyond the acoustic diffraction limit has been developed and continuously improved. With the help of unprecedented spatial resolution and reasonable temporal resolution, this technology could significantly enhance the diagnosis and monitoring of the diseases that are associated with abnormal changes of microvasculature. A number of preclinical studies have already demonstrated the feasibility in vivo on different models, including tumors, kidneys, brain imaging, etc. Overall, while some challenges exist for future clinical translation, SRU imaging technology still holds a great potential for broad clinical applications with a high impact.

## Figures and Tables

**Table 1:** The overall performances of the representative SRU imaging technologies. * All technologies offer spatial resolution beyond the acoustic diffraction limit.

	Localization [32]	SOFI [43]	Deconvolution [36]
Spatial Resolution *	High (9~17 µm)	Low (227.3 ± 9.0 µm)	Middle (41 µm)
**Numbers of Frames for Reconstruction**	75,000 frames	150 frames	300 frames
**Temporal Resolution**	150 s @ 500 Hz	0.3 s @ 500 Hz	0.6 s @ 500 Hz
**Microbubble Concentration**	Low (Diluted, 2 × 10^8^ MBs/mL, Bolus Injection of 1.5 mL)	High (1.2 × 10^10^ MBs/mL, Bolus Injection of 0.5 mL)	High (1.2 × 10^10^ MBs/mL, Bolus Injection of 0.2 mL)
**Application**	Brain	Kidney	Atherosclerosis, Kidney

**Table 2:** An overall comparison of the representative deep learning-based technologies currently used in SRU imaging. * Only in vivo data were used for training. The dosage of injected microbubble is 2.5 × 10^7^ MBs in 60 μL saline.

	Deep-ULM [39]	CNN Based Network for Multiple Target Detection [50]	mSPCN-ULM [40]	Deep 3D CNN for Spatiotemporal Filtering [51]
**Target**	Localization from the dense microbubbles	Localization from the dense microbubbles	Localization from the dense microbubbles	Microbubble extraction
**Microbubble Concentration of Synthetic Data for Training**	High (~2.6 MBs/${\mathrm{mm}}^{2}$)	High (~2.44 MBs/${\mathrm{mm}}^{2}$)	Very high (~6.4 MBs/${\mathrm{mm}}^{2}$)	N/A *
**Network Type**	U-net	Convolutional neural network	Modified subpixel convolutional neural network	3-D convolutional neural network
**Training Dataset**	Synthetic data and unique data generated for each iteration	10,240 synthetic data	10,000 synthetic data	9000 frames acquired from five subjects
**Spatial Resolution**	~30 μm	27~46 μm	24~28 μm	25 μm
**Applied Activation Function**	Leaky rectified linear unit (ReLU)	Leaky ReLU	ReLU	ReLU

## Data Availability

Not applicable.
